# Supplementary material for: Are carbon emissions trading and green financial instruments synergistic? -Comprehensive quantitative research based on content analysis
Source: PLoS One. 2024 Mar 7;19(3):e0298601. doi: 10.1371/journal.pone.0298601 (PMC10919601; doi:10.1371/journal.pone.0298601)
Supplement: S1 Data — (ZIP) [file pone.0298601.s001.zip › Data/地级市Z_F工作报告.zip!/║╙▒▒/║Γ╦«╩╨/└┤╫╘╣1⁄2╓┌║┼í╛┬φ┐╦╩2╛▌═°í┐_2014.docx]

各位代表：

现在，我代表市政府向大会作工作报告，请予审议，并请市政协委员和列席会议的同志提出意见。

一、2013年工作回顾

2013年是本届政府履职的第一年。面对复杂严峻的发展形势和愈加突出的大气污染防治压力，全市上下在省委、省政府和中共衡水市委的正确领导下，认真贯彻党的十八大和十八届二中、三中全会精神，按照“强势开局、跨越赶超，提前建成小康社会”总要求，深入实施“项目立市、工业强市、三产兴市、农业升级、绿色崛起”发展战略，全力以赴稳增长、调结构、抓改革、破瓶颈、优环境、惠民生，经济社会发展呈现“逆势上扬、跨越赶超，增速领先、总量晋位”的可喜局面，美丽新衡水建设迈出坚实步伐，较好地完成了市五届人大一次会议确定的目标任务。

全市实现生产总值1070.1亿元，比上年增长9.1%。全部财政收入129.1亿元，公共财政预算收入68.5亿元，分别增长28.1%和35.1%。全社会固定资产投资830.1亿元，固定资产投资787.3亿元，分别增长22.5%和23%。社会消费品零售总额485.8亿元，增长13.7%。规模以上工业增加值411.1亿元，增长12.1%。外商直接投资20166万美元，增长12.1%。各项存款余额1854.6亿元，增长18%。全部财政收入、公共财政预算收入、全社会固定资产投资、固定资产投资、规模以上工业增加值5项指标增速位居全省首位，固定资产投资、规模以上工业增加值、外商直接投资、各项存款余额4项指标实现总量晋位。10个县市财政收入全部超3亿元，景县、安平突破10亿元。

抓项目，促投入，经济发展有了新支撑。100项省市重点续建和新开工项目完成投资391亿元，100项省市重点前期项目有30项提前开工。亿元以上项目完成投资535.5亿元，增长35.2%，竣工亿元以上项目达到103项。老白干三期改造、泰纳改性合成橡胶新材料、吉瑞精密铸造、养元植物蛋白饮料扩产等重大项目投产或部分投产；威克多制衣、冀中能源矿用装备制造等加快推进；海伟丙烷脱氢、阳煤乙二醇开工建设，实现了单体百亿元产业项目“零”的突破。邯黄铁路建成通车，南水北调配套工程、邢衡高速、衡德高速故城支线等基础设施项目扎实推进。狠抓项目谋划论证和结构性评价，新谋划亿元以上项目1091项，计划总投资7703亿元；省市重点项目中高新技术、战略性新兴产业、装备制造和服务业项目占比达到60%，比上年提高10个百分点。

抓调整，促转型，产业结构出现新提升。深入实施“两改造一倍增”计划。完成技改投资363.1亿元，增长30.4%；列入省千项技改工程项目104项，获得省扶持项目和资金数均居全省首位；推进现代企业制度建设，围绕提高经营者素质和企业管理水平完成各类培训12.5万人次。实现“个转企”592家、“小转一”272家、“下转上”193家。纳税超千万元企业达到49家，养元公司、老白干集团纳税分别达到7.4亿元、4.6亿元，增长31.5%和20.2%。加快服务业拓展提升步伐。组建了市旅游局，成立了滨湖旅游有限公司，建成了马拉松广场二期、龙源酒店等旅游基础设施项目，衡水湖晋升4A级景区。完成货运周转量274.7亿吨公里，增长18%，安平国际丝网物流聚集区一期竣工投用，衡德物流园晋升省级物流产业聚集区。新增限额以上批零住餐单位244家，奥特莱斯项目开工建设，衡百国际正式开业。文化产业投资大幅增长，武强国际乐器文化产业基地入驻企业20余家，饶阳青铜雕塑艺术产业园、武邑硬木雕刻产业园投入运营。

抓规模，促流转，现代农业实现新突破。粮食总产37.25亿公斤，连续4年稳定在35亿公斤以上。以规模农业、法人农业、科技农业为抓手加快现代农业强市建设，全市流转土地面积达到212.5万亩，占家庭承包总面积的27.1%，提高10.5个百分点；建成3000亩以上规模经营典型38个；注册家庭农场895家，登记农民合作社8123家，均居全省首位。谋划实施千万元以上农业产业化重点项目135项，完成投资180亿元，华都、新发地等一批项目加快建设，新增省级重点龙头企业20家，预计农业产业化经营率达到61%，提高2.1个百分点。做好扶贫开发工作，9万贫困人口稳定脱贫。全省农村工作会议以现场会形式在我市召开，我市深化农村改革、发展现代农业工作得到省委、省政府充分肯定。

抓城建，促统筹，城乡面貌发生新变化。确立了“一湖三城”大衡水格局和城市组团发展思路，滨湖新城规划和招商工作提速推进，河东改造正式启动，南部新区土地收储圆满完成，城市要素聚集能力和增值效应明显提升。加强中心城市基础设施建设，投资2.2亿元实施供热一次网改扩建工程，可满足市区未来10年供热需求；铺设天然气路北环网18公里，实现了双线安全供气；滏阳新河深槽截污导污、滏东排河污水治理工程基本完工；实施13条街巷改造提升工程，榕花大街贯通工程建成通车，成为连接三区、贯通南北的重要通道。加快县城扩容升级，在建超千万元市政项目91个，完成投资35亿元。强力推进城镇环境质量、环境卫生、园林绿化等10个专项整治。扎实开展深化加强基层建设年活动，实施农村环境卫生和面貌改造提升行动，投资16.5亿元完成805个重点村改造任务，全市涌现出冀州北内漳、饶阳圣水村、桃城区北苏闸等一批升级版的现代农村。

抓治理，促保护，生态建设取得新成效。出台大气污染防治行动计划实施方案和一揽子政策措施，组织开展7个专项行动，关闭重污染企业227家，拆除分散燃煤供热锅炉132台，淘汰黄标车27798辆，规范治理市区露天烧烤157处，对市区117处在建项目、53处砂石料场和煤场进行了全面整治。购置天然气公交车217辆，在全省率先淘汰市内公交黄标车。购置环卫作业机械62部，机械化清扫率达到65%。实施燃煤锅炉改造、余压余热利用等十大节能工程，推进污水处理厂、垃圾处理场建设改造和电厂烟气脱硫脱硝等减排项目。组建公安环境安全保卫支队，加大环境污染违法犯罪打击力度。预计全市单位生产总值能耗下降3.43%，化学需氧量、二氧化硫、氨氮、氮氧化物均完成省下达的减排目标。以规模造林和通道绿化为重点，扎实推进“一人一亩林”工程，完成造林绿化29.2万亩，建成千亩以上林业园区7.03万亩。

抓民生，促和谐，人民生活有了新改善。全年财政用于民生支出133亿元，占公共财政预算支出的74.1%。承诺的十件民生实事基本完成。城镇居民人均可支配收入20541元，农民人均纯收入6975元，分别增长11%和13.1%。城镇新增就业3.9万人。社会保障水平继续稳步提高，企业退休人员养老金待遇人均月增182元，城乡低保标准分别提高14.4%和21.7%，新农合和城镇居民医疗保险年人均财政补助标准由240元提高到280元。新建农村互助幸福院635所，为85岁以上老人发放高龄补贴1500万元。全市856名孤儿全部纳入保障范围。开工建设保障性住房11281套，竣工8113套，刘里马、孙三村等城中村改造加快推进。新建扩建水厂15座，50万农村人口饮水安全问题得到解决。新建标准化村卫生室2849个。投资21亿元完成覆盖全市637所中小学的校舍安全工程，改造校舍277万平方米；市区康复街小学、第四中学改扩建竣工；衡水中学新校区、衡水学院西校区加紧建设。普通高中教育水平进一步提升，升入清华、北大等名牌大学人数稳居全省首位。成功举办第二届衡水湖国际马拉松赛并获全国金牌赛事，市区新增篮球活动场地22块，创建长跑城、篮球城活动扎实开展。“厚德衡水”主题道德实践活动不断深化。平安衡水建设深入实施，安全生产和食品药品安全监管力度加大，打击“两抢一盗”取得重大战果，社会大局和谐稳定。

抓改革，促开放，经济发展迸发新活力。在全省率先启动商事登记制度改革，工商注册登记增长25.1%。积极稳妥开展农村土地分类确权登记颁证试点工作。巩固实施基本药物制度，6个县级公立医院改革稳步推进。大力简政放权，分3批削减市本级行政审批事项30项，平均办理时限由13天压缩到7天。滨湖新区管理体制进一步理顺，工业新区“三区整合”步伐加快。举办2013衡水湖国际商务洽谈会等招商活动，全年签约项目168项，总投资2050亿元，引进资金1776亿元，招商引资开始由“走出去”、“请进来”向“找过来”转变。争进首都经济圈取得实质性进展，省已正式上报国家发改委，与北京铁路局、大兴区签订战略合作协议，中粮、首农、天士力、新兴际华等战略投资机构来我市洽谈，中国高新、中国铁物、恒大、居然之家等落户我市，“衡水湖号”客运专列顺利开通。3家企业在石交所上市，交通银行、河北银行、邢台银行入驻我市，金融机构存贷比提高2.9个百分点。对外贸易较快增长，实现出口总额32.1亿美元，增长18.5%，故城、枣强晋升国家外贸转型升级专业型示范基地。

抓作风，促落实，政府自身建设展现新形象。严格落实改进工作作风、密切联系群众各项规定，积极开展党的群众路线教育实践活动预热升温工作，大力开展正风肃纪专项行动，查处“吃空饷”人员2152人，处置违规公务车341辆，取消O牌车829辆，清理超标办公用房7.06万平方米，市直机关“三公”经费支出较上年压减5%。扎实推进市政府系统标准化建设，首批19个政府部门全部通过认证。自觉接受人大及其常委会的法律监督和工作监督、政协的民主监督以及社会监督，办理人大代表建议153件、政协提案276件，按时办结率均为100%。

注重加强国防与军队建设，国防动员能力持续提升，民兵预备役建设水平稳步提高，人民防空、双拥共建和优抚安置工作取得明显成效。人口计生、广播电视、新闻出版、妇女儿童、老龄、残疾人等事业持续发展。统计、档案、气象、邮政通信、民族宗教、外事侨务等工作都取得了新的成绩。

各位代表!一年的成绩来之不易。成绩的取得，是省委、省政府和市委正确领导的结果，是市人大、市政协监督支持的结果，是全市人民同心同德、奋力拼搏，社会各界和衷共济、通力协作的结果。在此，我代表市政府，向在全市各条战线辛勤工作的建设者、劳动者，向人大代表、政协委员，向老领导、老同志，向各民主党派、工商联、无党派人士、人民团体和社会各界朋友，向驻衡部队、武警官兵、政法干警和中省直驻衡各单位，向始终关心、关注家乡发展的优秀衡水儿女，致以崇高的敬意和衷心的感谢!

回顾一年来的工作，我们深刻体会到：必须坚持解放思想、坚定信心，深刻认识和准确把握我市发展所处方位，发挥区位优势、生态优势和农业优势，变隐性优势为显性优势、落后差距为后发优势、比较优势为竞争优势，努力走出一条差异竞争、特色发展的新路子。必须坚持开放引领发展、改革破解难题，自觉运用市场、开放、创新、法治的思维和办法推动科学发展，最大限度释放改革开放红利。必须坚持从我市实际出发，主动做好转方式调结构这道“必答题”，在加快赶超的同时更好地推动发展转型。必须坚持做正确的事，正确地做事，多做打基础、利长远、创环境、惠民生的实事好事，聚集改革发展正能量，形成政府与人民同心同向的生动局面。

各位代表，在看到成绩的同时，我们也清醒地认识到，我市经济社会发展还存在不少困难和问题，政府工作与人民群众期待还有差距。我市产业结构总体低端化的状况还没有根本改变，传统产业链条短，质量效益不高，创新驱动能力不足，新兴产业和服务业发展滞后。中心城区聚集度偏低，功能不完善。土地、资金、劳动力等瓶颈制约将成为常态，大气、水等生态红线约束更趋强化。政府职能转变和简政放权任务仍很艰巨，“门难进、脸难看、话难听、事难办”现象还没有从根本上杜绝，消极腐败现象还不同程度存在。我们一定高度重视人民群众的关切，直面自身工作中的问题，本着对党和人民高度负责的精神，以落实“八项规定”和反“四风”为切入点，采取更具针对性、更富突破性、更有实效性的措施大力加以解决，不辜负全市人民的期望!

二、2014年政府工作的指导思想和主要预期目标

2014年是贯彻落实党的十八届三中全会、省委八届六次全会和市委三届五次全会精神、全面深化改革的开局之年，是为圆满完成“十二五”规划目标奠定决定性基础的一年，也是我市强势开局、跨越赶超十分重要的一年。综合研判我市经济社会发展面临的形势，外部环境向好，内生动力增强，发展势头强劲，发展前景广阔。党的十八届三中全会拉开了全面深化改革的大幕，省委、省政府即将批复《衡水综合配套改革试验总体方案》，必将极大地激发我市经济社会发展活力；京津冀协同发展，环渤海地区加快发展，必将为我市在区域经济中发挥比较优势、推动融合对接提供历史性机遇；我市原有优势不断巩固，过去的劣势向优势转变，经济发展一年比一年好，发展成效一年比一年大，干部群众的信心和气势一年比一年足，随着党的群众路线教育实践活动深入开展，必将进一步巩固和发展全市风清气正、干事创业的良好氛围。我们要牢固树立机遇意识、进取意识、责任意识，坚持把发展作为第一要务，进一步凝聚跨越赶超、绿色崛起的共识，在正确的路径上踏石留印，在行之有效的举措上抓铁有痕，把市委三届五次全会绘制的宏伟蓝图落实在衡水大地上!

今年政府工作的指导思想是：认真学习习近平总书记系列重要讲话精神，全面贯彻落实党的十八大和十八届三中全会，中央经济工作会议、城镇化工作会议、农村工作会议，省委八届六次全会和市委三届五次全会精神，继续以“强势开局、跨越赶超，提前建成小康社会”为总要求，以综合配套改革为统领，着力推动产业转型升级，着力推进新型城镇化，着力建设现代农业强市，着力创优经济发展环境，坚定不移地走全面改革、绿色崛起、富民强市、普惠民生之路，把全面建成小康社会各项事业奋力推上新台阶。

综合考虑各方面因素，今年经济社会发展的主要预期目标是：生产总值增长9%左右；公共财政预算收入增长12%以上；全社会固定资产投资增长19%，固定资产投资增长20%；社会消费品零售总额增长13%；出口总额增长8%；实际利用外资增长5%；城镇居民人均可支配收入增长10%，农民人均纯收入增长11.5%；单位生产总值能耗下降3.43%，化学需氧量、氨氮、氮氧化物排放量分别削减1%、3.21%、8.1%，细颗粒物浓度下降4%；居民消费价格指数涨幅控制在3.5%左右；城镇登记失业率控制在4.5%以内；人口自然增长率控制在7.13‰以内。上述指标的提出，综合考虑了发展需要和支撑条件，与“十二五”规划、全面建成小康社会目标相衔接，体现了稳中求进、合理增长原则，也为全面深化改革、加快结构调整和生态环境治理创造条件、留出空间。

实现今年经济社会发展目标，必须在转变经济发展指导思想的基础上，突出“抓改革、打基础、造环境、求赶超”。抓改革，就是抓住综合配套改革试点市这一重大机遇，坚持把改革创新作为根本动力，贯穿于经济社会发展的全过程和各个方面，以改革破解瓶颈，以改革促转型升级、促民生改善。打基础，就是树立正确的发展观和政绩观，加强基础设施建设，推进基础产业发展，扎实做好各项基础性工作，增强发展的稳定性和持久性。造环境，就是强化环境是第一资源、生态是最大潜力的理念，创优发展环境，改善生态环境，赢得区域竞争的软实力和硬资本。求赶超，就是以提质、增效、升级为中心，千方百计促进经济持续快速健康发展，既稳扎稳打步步为营，又抢抓机遇争先晋位。

三、2014年主要工作安排

按照市委三届五次全会部署，2014年政府工作要以全面深化改革统领全局，重点做好以下八个方面的工作：

(一)着力推进投资和项目建设。坚定不移地把抓投资上项目作为首要任务，发挥投资第一拉动力的作用，努力在项目规模和质量上实现新突破。

突出抓好“1311”工程。全市安排省市重点项目100项，新开工亿元以上项目300项，谋划储备亿元以上项目1000项，确保竣工投产项目100项。把发展项目的基本着眼点放在市场自身发展的项目上，发挥项目在产业升级中的引领作用，突出抓好投资100亿元的华德新型清洁环保金属板、投资40亿元的泰华伟业机器人产业园、投资15亿元的亚森工业机器人、投资15亿元的中恒太阳能光热发电装备、投资12亿元的博得轨道交通设备、投资12亿元的澳泰节能制冷压缩机组、投资11亿元的麦格尼菲复合材料等产业项目。紧盯国家宏观政策导向，进一步加强项目谋划论证，对所有新上项目进行结构性评价，从源头抓起促进项目不断生成、科技不断创新、质量不断提高。

实施城乡基础设施提升行动。谋划实施一批具有基础性、全局性、战略性影响的重大项目，力争用3到5年时间，使全市经济社会发展基础条件得到明显改善。今年，积极推进石济客专、邢衡高速、衡德高速故城支线、大广高速衡水湖连接线等项目;创新体制，加大投入，加强县乡村公路改造养护工作，提高道路等级和通行能力。同时，推进衡水通用机场、石衡高速、京衡客专等项目前期，启动衡水机场前期研究工作。

放大县域经济优势，千方百计保障要素供应。实行差别化政策，建立指标安排与用地进度、供地率、补充耕地挂钩机制，重点向大项目、好项目倾斜。通过改革做好存量文章，提高土地利用率。支持企业上市融资，力争新增上市企业10家以上，实现县市区上市企业全覆盖;完善政银企合作机制，改善金融生态环境，推进符合条件的县级农村信用社改制为农村商业银行。

(二)着力建设现代农业强市。按照稳定政策、改革创新、持续发展的总要求，以确保粮食安全、稳定粮食生产、提升农业综合生产能力为目标，以土地流转为抓手，以规模农业、法人农业、科技农业、节水农业和现代农业示范区为突破点，努力在建设现代农业强市上走在全省前列。

编制现代农业发展规划。围绕农业强、农村美、农民富，立足于把我市建设成为全省现代农业先行区、循环农业示范区和首都经济圈重要的农产品加工聚集区，市县两级都要编制现代农业发展规划，有步骤、有计划、有重点地推进各项工作开展。

加快建设现代农业示范区。制定现代农业园区建设发展实施方案，从整合资源、拓展功能入手，加大对武强北大洼、故城东大洼、饶阳众悦、景县津龙等典型的支持力度，每个县市区都要抓一个以上现代农业园区，培育现代农业发展的示范区。

大力推进农业产业化经营。鼓励发展混合所有制龙头企业，引导农业龙头企业与合作社、农户有效对接，建立紧密型利益联结机制。大力发展现代食品加工业，抓好武强华都肉鸡产业一体化、故城正大农牧产业链、深州东方绿树果品深加工等重点项目建设，加强与中粮、天士力等战略投资者洽谈对接，力争农业产业化经营率提高1.5个百分点以上。调整农业结构，设施蔬菜发展到90万亩，商品猪出栏达到460万头，积极鼓励奶牛养殖业发展，确保年内奶牛存栏16万头。完善农产品批发市场信息网络和农村市场供求信息系统，坚持线上线下同步，为农村经济组织搭建对接国内外市场的平台。

抓好农产品质量和食品安全工作。按照中央“四个最严”的要求，严格落实属地管理责任和生产经营主体责任，进一步完善监管制度、强化监管手段，建立健全农产品安全标准、质量检测体系和农产品质量全程追溯体系，逐步形成覆盖从田间到餐桌全过程的监管制度，确保广大人民群众“舌尖上的安全”。

统筹抓好农业农村各项工作。以保障粮食安全和促进农民增收为核心，落实各项强农惠农富农政策，积极开展粮食高产创建活动。加快小型农田水利重点县建设，争列国家农业废弃物综合利用试点。推进农业科技进步，提高农业物质技术装备水平。以产业扶贫为核心，创新扶贫开发机制，改善农业农村生产生活条件，实现扶贫对象稳定脱贫。大力推进省市重点村面貌改造提升，全面打响厕所改建、饮水安全、道路硬化、垃圾处理、村庄绿化、危房改造六大战役，建设幸福家园和美丽乡村。

(三)着力推动工业转型升级。坚持无中生有、有中生新，改造提升传统产业和培育壮大战略性新兴产业双轮驱动，加快结构调整、转型升级、两化融合步伐，推动工业经济健康持续快速发展。

加强立市主导产业培育。立足现有特色产业，从理论和实践两个层面凝练提升，明确产业转型升级的战略方向，培育立市主导产业。依托工程橡胶打造铁路交通工程设施及装备制造业，依托丝网打造功能材料及制品制造业，依托养元和老白干等打造食品及生物制品制造业，依托玻璃钢打造节能环保及新能源产业，依托橡塑制品打造物料输储设施及装备制造业，依托铁塔钢构打造电力通讯设施及装备制造业。鼓励企业兼并重组，支持培育行业巨人，力争培育养元公司等企业实现销售收入超百亿元。

加强园区(开发区)建设。完善园区基础设施，积极探索整区招商等园区建设新机制，力争工业新区晋升国家级开发区，桃城经济开发区、大营经济开发区、武邑经济开发区、衡德工业园(北区)、深州化学工业园晋升省级园区。推进武强中外乐器文化产业园、龙华台湾工业园建设，力争建成特色鲜明的国别园。加快开发区内陆港建设，争取年内竣工运营。加快饶安经济协作区、运河新区等园区建设。

加强技术改造和管理改造。落实省工业企业技术改造升级计划，研究支持工业技改的工作方案和政策措施，争取列入省千项技改项目100项以上。继续组织实施“企业经营者素质提升计划”和“现代企业制度建设工程”，加强企业家队伍建设，带动企业管理水平提升和商业模式创新。实施创新驱动战略，发挥企业创新主体作用，从标准、研发、渠道等关键环节入手，推动产学研用结合，力争新增科技型中小企业500家、省级产业集群公共技术服务平台3家、中小企业创业辅导基地5个。

加强工业运行调节和政策支持。完善“订单式”服务制度，推进“1+10”行动计划，每个产业筛选10家重点企业予以点对点支持。积极争列省“三个一百”工程。加快推进“个转企”、“小转一”、“下转上”，力争新增规模以上企业150家。完善国有资产监督管理体制，确保国有资产安全，提高国有资产效率。

(四)着力加强城市建设管理。坚持扩容与提质并重，高起点做好规划设计，不断提升城市建设管理水平，打造生态湖城、美丽衡水，努力建设融入首都经济圈、面向环渤海的冀东南区域中心城市。

优化城乡空间布局。坚持城乡一体，深化规划改革，创新规划管理，构建梯次衔接的城镇化规划体系。划定中心城区和县城开发边界，使城市规划逐步由扩张性规划向限定城市边界、优化城市空间结构的规划转变。推进中心城市“一湖三城”组团式发展。各县市按照“小县大县城”战略进行规划，建设宜居宜业的中小城市。切实发挥乡镇积极性，力争每个乡镇都成为城镇化的亮点。积极稳妥推进农村新型社区建设。选择有产业基础、有文化底蕴的村优先支持，建设美丽新农村。编制完成全市城乡总体规划及道路交通、市政设施等专项规划，编制完成10个县城控制性详细规划。

提升城市功能品位。启动榕花大街、永兴东路、南外环等街道改造提升工程，打通庆丰街、宝云街等城市断头路，抓好胡堂排干截污导流、前进街污水治理工程，实施垃圾池改造提升、城区公厕、停车场、菜市场、街头游园、小街小巷、裸露地面覆盖等城市功能完善工程。抓好桃城区南部新区建设和河东老城区改造，积极推进城中村改造。以综合改革的思路推动滨湖新城建设，统筹推进安置社区建设、基础设施建设、旧村拆迁、土地收储和社会保障等。实施工业新区基础设施综合提升工程，谋划实施高铁新区建设，提高新区承载能力。坚持拆旧与建新相结合，大力推进县城城中村拆迁改造，组织实施一批县城基础设施建设工程。冀州、深州、安平、景县、故城、枣强要努力向中等城市目标迈进，其他县城要打造精品小城市。

创新城市运营管理。整合城市管理资源，理顺管理体制，推行综合执法，解决好错位、越位、不到位的问题。延伸城管触角，加强社区建设，提高社区自治和服务功能，提升小区物业管理水平。开展城市环境综合治理，加大治违、治脏、治乱力度，积极开展创建国家园林城市活动，把生态治理与美化城市结合起来，构建优美和谐的人居环境。

(五)着力提升服务业发展水平。把服务业作为落实转方式调结构，实现跨越赶超、绿色崛起的重要方向和抓手，坚持扩大总量与优化结构并重，生产性服务业与生活性服务业并举，促进服务业发展提速、比重提高、水平提升。

加快发展现代物流业。围绕首都经济圈南部物流中心定位，争列国家和省物流产业发展规划，抓好与北京铁路局、省建投等战略合作协议落实，依托工业新区谋划建设现代物流园区。积极推进新发地农副产品物流园、安平内陆港、安华国际物流园、衡德商贸综合物流等项目建设。加快构建综合运输体系，调整运力结构和运输组织方式，提升运输效率和效益。引入现代管理理念和新型商业模式，支持电商服务平台建设，抓好桃城橡胶、安平丝网、大营裘皮、景县胶管、冀州暖气片、武邑金属橱柜等市场改造和电子商务交易平台建设，鼓励大型商贸企业建设网上商城和中小微企业开设网店。

大力发展文化旅游业。强化旅游品牌意识，以衡水湖旅游为龙头，推进精品景区、精品线路建设，打造独具特色的旅游休闲目的地。促进文化旅游融合发展，深入挖掘冀文化、儒文化、红色文化资源，抓好周窝音乐小镇、衡水湖文化产业聚集区、董子故里、冀州古城恢复、老盐河开发等重点文化项目建设。深化文化体制改革，培育文化市场主体，引进一批知名文化品牌，发展一批文化创意骨干企业，建设一批文化旅游名城名镇名村。

壮大提升商贸流通业。打造京南特色商贸名城，启动隆基泰和、深圳茂业、新天地等城市综合体项目，加强城乡便民商贸网点建设，提升商贸流通、餐饮购物等传统服务业，改善消费环境。在主城区打造特色商业街区、商贸功能区、中央商务区，各县市培育1-2个商贸功能区和特色商业街区。

(六)着力扩大对外开放。坚持开放引领发展，以招商引资为核心，拓展开放领域，提高开放水平。

创新招商方式。在积极参加全省招商活动的同时，提升衡水湖国际商务洽谈会影响力。建立常态化、市场化、专业化招商机制和队伍，继续抓好定向招商。围绕培育主导产业，绘制产业链图谱，制定专项招商行动方案，瞄准重点区域，紧盯行业龙头，拓展定向招商范围，争取在引进大集团、大企业上实现新突破。大力引进战略投资者，力争每个县市区都有50亿元、100亿元大项目、好项目落地。

强攻重点招商区域。抓住京津功能疏散、产业转移和京津冀战略合作机遇，全面开展对接京津活动，争取首农婴幼儿奶粉等项目早日落地。继续加大与珠三角、长三角经济技术合作力度，利用好上海自贸区窗口，扩大衡水“走出去”步伐。

培育壮大出口主体。抓好安平、故城、枣强国家外贸转型升级专业型示范基地建设。强化外贸企业分类帮扶，实施以质取胜和市场多元化战略，积极为出口企业搭建服务平台，力争出口备案企业新增100家以上，培育出口超亿美元企业。

(七)着力推进生态治理和造林绿化。把生态建设放在关系发展全局的战略高度，坚决打赢大气污染治理攻坚战、持久战，进一步塑造、培育和拓展我市的生态优势。

铁腕治理大气污染。严格执行14条防治措施，深入开展7个专项行动，从严查处各种环境违法行为，力争空气环境质量3年有好转、5年大改善。继续淘汰黄标车。严格控制扬尘污染，严控煤炭消费总量，全年完成11.2万吨煤炭削减任务。广泛推广农村清洁能源应用，实施农村取暖治理提升行动，全面禁止秸秆焚烧，减少燃煤和面源污染。落实重污染天气应急预案，切实减轻重污染天气对群众生产生活和身体健康的影响。加强与周边地区协作，推进大气污染治理联防联控。倡导绿色生活方式，建立大气污染治理公众参与机制，营造“爱护环境、从我做起”的浓厚氛围。

强力推进节能减排。强化能源消费总量控制和燃煤减量替代约束，组织实施节能项目10项以上，力争形成年节能能力6万吨标煤。抓好污水处理厂建设工程，赵圈镇、龙华镇和工业新区污水处理厂4月份建成投用，其他污水处理厂建设改造项目年底前投入运行。完成冀州中科能源有限公司烟气脱硝工程。

切实加强地下水超采综合治理。把地下水超采治理放在与大气环境污染治理同等重要的位置，全力以赴抓好国家级地下水超采综合治理试点工作，力争走在全国全省前列。按照国家和省部署，围绕调整种植模式、加强水利建设、创新体制机制、严格控制超采等重点，科学制定综合治理规划和年度实施方案，加紧推进项目前期准备工作，最大限度争取国家和省支持。抓好水环境治理，确保衡水湖水质稳定达到III类标准，滏阳河等重点流域达到省政府考核标准，全市其他重点河渠也要达到相应的水质标准。完成南水北调配套工程，使国家工程惠及千家万户。

大力开展造林绿化。继续推进“一人一亩林”工程，完成造林16万亩。按照大功能区规划理念，突出环衡水湖区域和通道绿化，滨湖新区、桃城区、冀州、枣强、武邑、深州重点抓好衡水湖森林公园、枣强森林公园等6-10个万亩以上生态片林，打造环湖生态隆起带。抓好以高速公路、国道为重点的主干道通道绿化百米拓宽工程。稳步扩大经济林比重。坚持抓大不放小，统筹推进各县市区造林绿化，做好省市重点村的绿化美化。

(八)着力保障和改善民生。把促进社会公平正义、增进民生福祉作为政府工作的出发点和落脚点，立足当前办好具体实事，着眼长远注重制度安排，解决好人民群众最关心最直接最现实的利益问题。

加强就业和社会保障工作。健全城乡均等公共就业创业服务体系，完善扶持创业优惠政策，创造更多就业岗位，统筹做好高校毕业生、农村转移劳动力、城镇困难人员、退役军人等重点人员就业，推动实现更高质量就业。年内城镇新增就业3.5万人，新增农村劳动力转移就业2.8万人。完善社会保障制度，提高企业退休人员基本养老金和城乡居民社会养老保险基础养老金水平，城乡居民医疗补助标准年人均提高到320元，新农合筹资标准提高到390元。扩大社会救助范围，提高社会救助标准，城市低保标准由每月347元提高到450元，农村低保标准由每年2120元提高到2500元，五保集中供养能力达到65%以上，加强农村互助幸福院运行管理。开工建设保障性住房19808套、竣工7500套，抓好农村危房改造。实施农村安全饮水工程，解决35万农村人口饮水安全问题。

促进社会事业全面进步。将市区中小学建设纳入城市发展规划，支持桃城区中小学建设，缓解班容量过大问题，重点推进前进街小学、东方太阳城小学新建工程和新苑小学、胜利小学、第十中学改扩建工程。继续支持衡水中学、衡水二中等重点高中发展，努力将基础教育优势转化为发展优势。深化校企合作，提高职业院校服务地方发展的能力和水平。加快衡水学院西校区建设，确保顺利通过教育部评估。加强城市社区卫生服务中心建设，继续推进县级公立医院综合改革，实行药品零差率销售，加强药品网上集中采购和电子监管，抓好重大疾病和传染病防控。鼓励社会资本进入健康产业、社会办医。建立贫困重度残疾人生活补贴和贫困残疾儿童抢救性康复补贴制度。加强文物和非物质文化遗产保护，支持文化精品创作，发展公共文化事业。办好衡水湖国际马拉松赛，完善城乡公共体育设施，谋划建设市全民健身中心，继续打造长跑城、篮球城。

深入推进平安衡水建设。牢固树立安全发展理念和安全生产常态化思维，深入实施“打非治违”、管道管网安全等专项治理，加强重点行业领域安全监管，坚决遏制重特大安全事故。深入开展食品药品隐患排查和专项整治，实施食品药品安全县创建工作。加强突发公共事件应急预警，扎实做好信访工作，严厉打击违法犯罪活动，努力保障人民安居乐业、社会安定有序。继续支持国防和军队建设，加大国防后备力量和国防动员建设力度，做好双拥共建工作，推进军民融合深度发展。支持工会、共青团、妇联等人民团体发挥桥梁纽带作用，做好新闻出版广电、老龄妇女儿童、气象防震减灾、民族宗教、人民防空、邮政通信、统计、档案方志、外事侨务和涉台事务等工作。

四、全面深化综合配套改革

今年是新一轮改革的开局之年。我市作为全省综合配套改革试点市，必须争当改革排头兵，以改革先行一步推动发展快赶几步。我们将按照中央和省委、省政府统一部署，制定实施综合配套改革试验总体方案，坚持问题导向，从制约经济社会发展最突出的问题改起，从老百姓最期盼的领域改起，从各方面能够达成共识的环节改起，推出一些“解渴”、管用的改革，让老百姓得到实实在在的好处，让全社会感受到市场环境、创业条件一天天在好转。重点在以下六个方面先行一步。

(一)在深化农村综合改革上先行一步。坚持社会主义市场经济改革方向，在明确底线的前提下，鼓励探索创新，支持先行先试，激发农村经济社会活力。在落实农村土地集体所有权的基础上，稳定农户承包权、放活土地经营权，加快健全土地经营权流转市场，完善县乡村三级服务网络和保障平台，引导土地有序流转，推进多种形式的规模经营。积极培育3000亩以上规模化经营典型，发展专业大户、家庭农场、农民合作社和农业企业等新型农业经营主体，支持工商资本到农村发展适合企业化经营的现代种养业和现代农业园区，进一步提高土地流转比例。大力发展农村土地合作社，在农民自愿、保证和提高农民土地承包权收益的基础上，最大限度地将无能力耕种或不愿耕种的家庭土地整合起来，组织开展农业招商，实行规模化经营。建立县级农村产权交易市场，促进农村产权公开规范交易流转。在保障农户宅基地用益物权前提下，慎重稳妥推进农民住房财产权抵押、担保、转让。加快供销合作社改革发展，努力把供销合作社打造成为农民生产生活服务的综合平台。

(二)在激活市场主体上先行一步。加快构建有利于全民创业的体制机制，毫不动摇地鼓励和支持民营经济发展，培育和壮大各类市场主体。继续深化商事登记制度改革，除少数特殊行业外，一律由“先证后照”改为“先照后证”，把注册资本“实缴”制改为“认缴”制，放宽市场主体注册资本、住所登记条件，把企业年检制度改为年报公示制度。支持衡水学院、衡水职业技术学院等有条件的单位建立创业基地。坚持权利平等、机会平等、规则平等，支持民间资本进入基础设施、基础产业和公用事业领域，大力发展混合所有制经济，为民营经济营造稳定、透明、公平的投资环境。力争到“十二五”末，万人市场主体由现在的292个增加到500个以上。

(三)在简政放权上先行一步。围绕打造“全省最优、全国一流”发展环境，加快转变政府职能，切实把发展经济的着力点转到为各类市场主体创造统一开放、竞争有序的市场环境上来。承接和落实好国务院、省取消和下放的行政审批事项，制定并公开行政审批事项目录，没有进入目录的一律停止审批。所有涉审部门都要成立项目领办服务中心，落实领办、代办等便民措施。加强各级政务服务中心建设，推进企业注册“一口清”、项目建设“一号通”、企业帮办“一卡通”。

(四)在增强县乡活力上先行一步。省下放到市的审批事项一律下放到县。以扩权强镇(乡)为导向，调动乡镇的积极性，发挥乡镇所在地在推进农村“四化”中的生力军作用。首批抓好龙华、大营、赵圈等经济强镇放权试点和镇区合一管理体制。凡是用新的办法解决问题、攻克难点，都是改革创新，都要大力支持。

(五)在优化金融环境上先行一步。抓住国家加快金融改革的机遇，放宽金融行业准入，支持具备条件的民间资本依法发起设立中小型银行、金融租赁公司和消费金融公司，鼓励民间资本投资入股金融机构和参与金融机构重组改造，支持金融机构到农村增设网点，让金融市场主体尽快多起来、活起来。支持发展各类投资企业，参与政府公共建设、市场企业发展的投融资。

(六)在推进新型城镇化上先行一步。把推进城镇化作为重大战略任务，努力走出一条以人为本、优化布局、生态文明、传承文化、具有衡水特色的新型城镇化道路。顺应社会发展客观规律和农民意愿，把有序推进农业转移人口市民化作为首要任务，推动农村人口向城镇集中。加快户籍制度配套改革。把进城落户农民纳入城镇住房和社会保障体系，稳步推进城镇基本公共服务常住人口全覆盖。建立农村劳动力转移就业实名制动态管理制度，鼓励农民工参加城镇职工基本医疗保险，允许灵活就业农民工参加当地城镇居民基本医疗保险。将农民工及其随迁家属纳入社区医疗卫生体系，免费提供健康教育、妇幼保健等公共卫生服务。

五、切实加强政府自身建设

各位代表，全面完成今年的目标任务，需要保持奋发向上的精神状态和求真务实的工作作风。我们将以开展党的群众路线教育实践活动为契机，不断提高各级政府的公信力和执行力，为强势开局、跨越赶超提供有力保障。

(一)勇于改革，充分激发创新活力。把全面深化改革作为破解发展难题的金钥匙，坚持解放思想，自觉运用市场思维、开放思维、创新思维和法治思维，靠改革寻出路找办法，以思想的大解放推动改革的大突破，促进发展的大跨越。把勇于担当作为奉献改革的精神追求，凡是有利于科学发展、改善民生、创造财富的措施和办法，都大胆试、大胆闯，绝不坐而论道、左顾右盼、畏首畏尾。

(二)转变职能，着力提高服务水平。强化政府公共服务、市场监管、社会管理、环境保护等职责，加强发展战略、规划、政策、标准等制定和实施。选择部分市直部门、服务项目作为试点，推行政府向社会力量购买服务。健全决策机制和程序，以群众利益、群众需求作为决策的基本出发点，认真听取各方面对政府工作的意见和建议。加强调查研究，察实情、出实招、办实事，总结基层经验，推动工作落实。继续推进政府机关标准化建设工作，提高政府服务基层、企业、群众、来衡投资者的针对性和精准度，提升工作节奏和服务水平。

(三)依法行政，努力建设法治政府。严格按照法定权限和程序行使权力、履行职责。坚决执行人大及其常委会决议决定，自觉接受其法律和工作监督，主动接受政协的民主监督，认真办理人大代表建议和政协提案，广泛听取民主党派、工商联、无党派人士和人民团体意见。加强行政监察、审计监督和行政层级监督，虚心接受社会监督和舆论监督。加强行政复议工作，完善依法行政考核评价制度，强化行政执法责任和过错追究。深化政务公开，让人民监督权力，让权力在阳光下运行。

(四)改进作风，不断提升行政效能。始终保持奋发有为的工作热情，始终保持蓬勃朝气、昂扬锐气，把目标定得更高一些，把眼界放得更宽一些，把劲头鼓得更足一些，带着激情、责任、追求，自觉把心思用在谋发展上，把精力用在干实事上，不断创造经得起实践、群众和历史检验的业绩。扎实开展党的群众路线教育实践活动，自觉抵制“四风”，严格落实“八项规定”，坚持领导带头、依靠群众、注重实效，从解决具体问题做起，切实将教育实践活动成效转化为惠民利民成果。

(五)从严治政，切实加强廉政建设。厉行勤俭节约，反对铺张浪费，加快预算管理制度改革，规范预决算公开内容、程序和形式，严控“三公”经费支出，大幅降低行政成本。深入推进政府系统惩治和预防腐败体系建设，严格落实党风廉政建设责任制，加强对权力运行的制约和监督，加大违法违纪案件查办力度，教育引导各级干部进一步增强廉洁自律意识，努力做到干部清正、政府清廉、政治清明。

我们的政府是人民的政府。我们将始终把人民是否满意作为检验评判工作的第一标准，以改革创新的精神不断加强自身建设，全面履行政府职责，用人民赋予的权力为人民谋福祉，努力建设人民满意的政府。

各位代表!人民对美好生活的向往是我们的奋斗目标，跨越赶超、绿色崛起是我们的崇高使命。让我们紧密团结在以习近平同志为总书记的党中央周围，在省委、省政府和中共衡水市委的坚强领导下，紧紧依靠全市人民，凝心聚力，真抓实干，为建设美丽新衡水、提前建成小康社会而努力奋斗!
